# Detection of Internal Hemorrhage via Sequential Inference: An In Silico Feasibility Study

**DOI:** 10.3390/diagnostics14171970

**Published:** 2024-09-06

**Authors:** Yekanth Ram Chalumuri, Xin Jin, Ali Tivay, Jin-Oh Hahn

**Affiliations:** Department of Mechanical Engineering, University of Maryland, College Park, MD 20742, USA; yekanth.ram@gmail.com (Y.R.C.); xjin9891@gmail.com (X.J.); ali.tivay@gmail.com (A.T.)

**Keywords:** hemorrhage, detection, sequential inference, observer, Kalman filter, virtual patient

## Abstract

This paper investigates the feasibility of detecting and estimating the rate of internal hemorrhage based on continuous noninvasive hematocrit measurement. A unique challenge in hematocrit-based hemorrhage detection is that hematocrit decreases in response to hemorrhage and resuscitation with fluids, which makes hemorrhage detection during resuscitation challenging. We developed two sequential inference algorithms for detection of internal hemorrhage based on the Luenberger observer and the extended Kalman filter. The sequential inference algorithms use fluid resuscitation dose and hematocrit measurement as inputs to generate signatures to enable detection of internal hemorrhage. In the case of the extended Kalman filter, the signature is nothing but inferred hemorrhage rate, which allows it to also estimate internal hemorrhage rate. We evaluated the proof-of-concept of these algorithms based on in silico evaluation in 100 virtual patients subject to diverse hemorrhage and resuscitation rates. The results showed that the sequential inference algorithms outperformed naïve internal hemorrhage detection based on the decrease in hematocrit when hematocrit noise level was 1% (average F1 score: Luenberger observer 0.80; extended Kalman filter 0.76; hematocrit 0.59). Relative to the Luenberger observer, the extended Kalman filter demonstrated comparable internal hemorrhage detection performance and superior accuracy in estimating the hemorrhage rate. The analysis of the dependence of the sequential inference algorithms on measurement noise and plant parametric uncertainty showed that small (≤1%) hematocrit noise level and personalization of sequential inference algorithms may enable continuous noninvasive detection of internal hemorrhage and estimation of its rate.

## 1. Introduction

Hemorrhage is accountable for approximately 40% of mortality in the world [1]. In the civilian sector, hemorrhage is the most common cause of deaths in young adults and juveniles [2]. In the military sector, >85% of mortality on the battlefield is attributed primarily to hemorrhage, 25% of which may be survivable if timely treatment is provided [3].

Hemorrhage can be external or internal. External bleeding can be easily detected and controlled. However, early detection of internal bleeding is not trivial. Rudimentary vital signs used in patient monitoring (e.g., heart rate (HR), blood pressure (BP), and oxygen saturation) do not reveal easily recognizable symptoms of bleeding and the resulting hypovolemia in the early stage of hemorrhage due to the body’s autonomic-cardiac compensation mechanisms [4,5,6]. Heart rate variability (HRV) is known to decrease during hemorrhage. But prior work showed that (i) it is a poor indicator of tolerance to hypovolemia [7] and (ii) it does not add value to rudimentary vital signs in identifying hemorrhage in patients receiving packed RBC transfusion [8]. Clinical efficacy of noninvasive measurements such as thoracic electrical bioimpedance, serum lactate, and mucosal pH has yet to be established [5]. Hence, early detection of internal hemorrhage before its recognition via obvious symptoms in rudimentary vital signs and other noninvasive measurements is important in providing life-saving interventions to hemorrhaging patients.

Efforts to promptly detect internal hemorrhage and the resulting circulatory decompensation have been made on both algorithmic and sensing fronts. Existing work on the algorithmic front includes pulse wave analysis (PWA) [9] as well as machine-learning-based methods to detect the depletion of blood volume (BV) [10] and compensatory reserve [4]. A potential weakness of these methods is that they are empiric and data-driven and, thus, are not readily interpretable. Such weakness raises concerns regarding the efficacy of these methods beyond the datasets used to develop them. In addition, these methods are often concerned with the inference of margins to circulatory collapse rather than the detection of hemorrhage itself, which is a disadvantage if the detection of hemorrhage itself is the main goal. Existing work on the sensing front includes imaging-based techniques and continuous blood hemoglobin (Hgb) monitoring (which is closely related to blood hematocrit (HCT), i.e., HCT is approximately 3 times Hgb [11]). Ultrasound imaging of inferior vena cava diameter and left ventricle thickness was ineffective in detecting hemorrhage [12], while computed tomography imaging and diffuse optical techniques showed promise in early detection of hemorrhage in pelvic injury [13]. But imaging-based techniques clearly have weaknesses in terms of ubiquitous usability in low-resource environments (e.g., prehospital settings as well as battlefield and mass casualty scenarios) due to the bulkiness of the equipment and the operator requirements. In addition, some imaging techniques require a priori knowledge of injury to accurately diagnose bleeding. Continuous Hgb monitoring based on pulse co-oximetry [14] showed promise in trending blood Hgb (and, accordingly, HCT). However, continuous Hgb monitoring is associated with substantial sensor noise [15]. In addition, Hgb may be associated with limited effectiveness in detecting internal hemorrhage in the presence of resuscitation with fluids because hemorrhage and fluids both dilute the blood and, thus, decrease Hgb. In sum, technology that can ubiquitously, promptly, and accurately detect internal hemorrhage does not appear to exist.

To bridge this gap, this paper investigates the feasibility of detecting internal hemorrhage and estimating its rate based on continuous noninvasive hematocrit measurement. Toward this goal, we developed two sequential inference algorithms for detection of internal hemorrhage based on the Luenberger observer (LO) and the extended Kalman filter (EKF). These algorithms exploit fluid resuscitation dose and hematocrit measurement as inputs to generate signatures to detect internal hemorrhage. In the case of the EKF, the signature is nothing but inferred hemorrhage rate, which allows it to also estimate internal hemorrhage rate. We evaluated the proof-of-concept of detecting internal hemorrhage and estimating its rate via these sequential inference algorithms by conducting in silico evaluation in 100 virtual patients subject to diverse hemorrhage and resuscitation rates.

This paper is organized as follows. Section 2 describes the plant dynamics, the design of linear and nonlinear sequential inference algorithms for detection of internal hemorrhage and estimation of its rate, an LO and an EKF, and the details of in silico evaluation of the sequential inference algorithms. Section 3 summarizes results, which are discussed in Section 4. Section 5 concludes the paper with possible future directions.

## 2. Methods

In Section 2.1, we present a lumped-parameter mathematical model of BV kinetics, which is the basis of designing an LO and an EKF for internal hemorrhage detection. In Section 2.2, we present novel internal hemorrhage detection algorithms based on an LO (Section 2.2.1) and an EKF (Section 2.2.2). In Section 2.3, we present the in silico evaluation method to evaluate the internal hemorrhage detection algorithms, including the virtual patients and scenarios (Section 2.3.1), the application of the algorithms to the in silico scenarios (Section 2.3.2), and the evaluation metrics (Section 2.3.3).

### 2.1. Plant Dynamics

**Figure 1:**
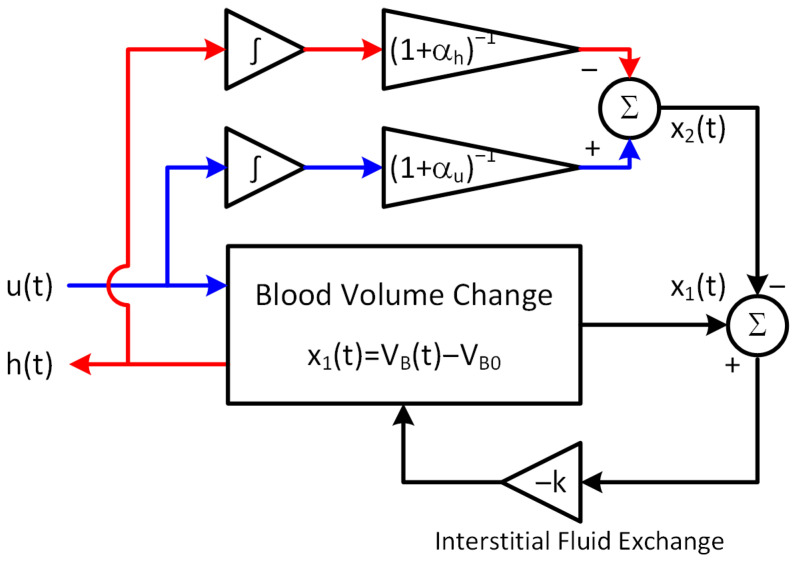
A lumped-parameter mathematical model of blood volume kinetics.

Most existing mathematical models of BV kinetics are extremely complex and involve a large number of individual-specific parameters [16,17,18,19,20]. Hence, these mathematical models may not be ideally suited to the design of observers and Kalman filters. For this reason, we employed a lumped-parameter mathematical model of BV kinetics developed in our prior work (Figure 1). A major strength of this mathematical model is that it can replicate the change in BV and HCT in response to hemorrhage and resuscitation while abstracting complex physiological details into simple phenomenological functions.

The lumped-parameter mathematical model is given by [21]:

(1) $$\left[\begin{array}{c} {\dot{x}}_{1}\left(t\right)\\ {\dot{x}}_{2}\left(t\right) \end{array}\right]=\left[\begin{array}{cc} -k & k\\ 0 & 0 \end{array}\right]\left[\begin{array}{c} x_{1}\left(t\right)\\ x_{2}\left(t\right) \end{array}\right]+\left[\begin{array}{c} 1\\ \frac{1}{1+\alpha_{u}} \end{array}\right]u\left(t\right)-\left[\begin{array}{c} 1\\ \frac{1}{1+\alpha_{h}} \end{array}\right]h\left(t\right)$$

where $x_{1}\left(t\right)$ is the change in BV from its initial value $V_{B0}$, $x_{2}\left(t\right)$ is the target change in BV, $u\left(t\right)$ and $h\left(t\right)$ are resuscitation and hemorrhage rates, respectively, and $\sigma \left(t\right)$ is HCT. The lumped-parameter mathematical model contains four interpretable parameters $\theta =\left\{V_{B0},\alpha_{u},\alpha_{h},K\right\}$, (i) $V_{B0}$ representing initial (i.e., pre-hemorrhage/resuscitation) BV; (ii) $\alpha_{u}$ and $\alpha_{h}$ representing the fraction of resuscitation and hemorrhage compensated by the interstitial fluid exchange (e.g., $\frac{1}{1+\alpha_{u}}$ fraction of resuscitation volume expands BV, while the remaining $\frac{\alpha_{u}}{1+\alpha_{u}}$ fraction is shifted to the interstitial space); and (iii) k representing the rate of interstitial fluid exchange.

In this work, we used two alternative output equations. For the LO, we used the following output equation:

(2) $$y\left(t\right)=\frac{\sigma \left(0\right)-\sigma \left(t\right)}{\sigma \left(t\right)}=\frac{1}{V_{B0}}x_{1}\left(t\right)+\frac{\int_{0}^{t}h\left(\tau \right)\sigma \left(\tau \right)d\tau }{V_{B0}\sigma \left(t\right)}$$

where HCT is assumed to be continuously measured, e.g., via continuous SpHb sensing [22,23]. Note that $y\left(t\right)$ includes the influence of both state (i.e., $x_{1}\left(t\right)$) and hemorrhage $h\left(t\right)$. In the absence of hemorrhage, $h\left(t\right)=0$ and $\frac{\int_{0}^{t}h\left(\tau \right)\sigma \left(\tau \right)d\tau }{V_{B0}\sigma \left(t\right)}=0$, which makes it possible to infer the fractional change in BV from $y\left(t\right)$: $y\left(t\right)=\frac{\sigma \left(0\right)-\sigma \left(t\right)}{\sigma \left(t\right)}=\frac{1}{V_{B0}}x_{1}\left(t\right)$. However, in the presence of hemorrhage, the term $\frac{\int_{0}^{t}h\left(\tau \right)\sigma \left(\tau \right)d\tau }{V_{B0}\sigma \left(t\right)}$ cannot be calculated because $h\left(t\right)$ is unknown in real-world settings. Hence, $y\left(t\right)$ is not a measure of the fractional change in BV anymore when $h\left(t\right)\neq 0$. For the EKF, we used the following output equation:

(3) $$y\left(t\right)=\sigma \left(t\right)$$

### 2.2. Internal Hemorrhage Detection via Sequential Inference

We developed two alternative sequential inference algorithms based on the LO and the EKF for detection of internal hemorrhage.

#### 2.2.1. Luenberger Observer (LO)

To design an LO, we augmented $h\left(t\right)$ as an additional slowly varying state to Equation (1). Then, the resulting state–space representation is given by Equation (4):

(4) $$\begin{array}{c} \dot{x}\left(t\right)=Ax\left(t\right)+Bu\left(t\right)=\left[\begin{array}{ccc} -k & k & -1\\ 0 & 0 & -\frac{1}{1+\alpha_{h}}\\ 0 & 0 & 0 \end{array}\right]\left[\begin{array}{c} x_{1}\left(t\right)\\ x_{2}\left(t\right)\\ x_{3}\left(t\right) \end{array}\right]+\left[\begin{array}{c} \begin{array}{c} 1\\ \frac{1}{1+\alpha_{u}} \end{array}\\ 0 \end{array}\right]u\left(t\right)\\ y\left(t\right)=\frac{\sigma \left(0\right)-\sigma \left(t\right)}{\sigma \left(t\right)}=\frac{1}{V_{B0}}x_{1}\left(t\right)+\frac{\int_{0}^{t}h\left(\tau \right)\sigma \left(\tau \right)d\tau }{V_{B0}\sigma \left(t\right)} \end{array}$$

where $A=\bar{A}+\tilde{A}$ and $B=\bar{B}+\tilde{B}$, with the superscripts $\bar{\cdot }$ and $\tilde{\cdot }$ being nominal and uncertain parts, respectively. Note that the output $y\left(t\right)$ does not represent the fractional change in BV when $h\left(t\right)$ is not zero, as described in Section 2.1. As outlined below, the LO neglects the integral term $\frac{\int_{0}^{t}h\left(\tau \right)\sigma \left(\tau \right)d\tau }{V_{B0}\sigma \left(t\right)}$ in its output equation and exploits the error caused by the neglected integral term to derive a signature of internal hemorrhage. The LO for the above plant dynamics is given by:

(5) $$\begin{array}{cc} \dot{\hat{x}}\left(t\right) & =\bar{A}\hat{x}\left(t\right)+\bar{B}u\left(t\right)+L\left[y\left(t\right)-\hat{y}\left(t\right)\right]\\ & =\bar{A}\hat{x}\left(t\right)+\bar{B}u\left(t\right)+L\left[\frac{\sigma \left(0\right)-\sigma \left(t\right)}{\sigma \left(t\right)}-\frac{1}{{\bar{V}}_{B0}}{\hat{x}}_{1}\left(t\right)\right] \end{array}$$

where L is the LO gain matrix. Note that we assumed that $\hat{y}\left(t\right)=\frac{1}{{\bar{V}}_{B0}}{\hat{x}}_{1}\left(t\right)$, i.e., we neglected the integral term $\frac{\int_{0}^{t}h\left(\tau \right)\sigma \left(\tau \right)d\tau }{V_{B0}\sigma \left(t\right)}$ in the output equation in Equation (4). The corresponding LO error dynamics are given by:

(6) $$\begin{array}{cc} \dot{e}\left(t\right) & =\dot{x}\left(t\right)-\dot{\hat{x}}\left(t\right)\\ & =\bar{A}e\left(t\right)-\frac{L\left[\begin{array}{ccc} 1 & 0 & 0 \end{array}\right]}{{\bar{V}}_{B0}}e\left(t\right)+\tilde{A}x\left(t\right)+\tilde{B}u\left(t\right)-\frac{L}{{\tilde{V}}_{B0}}x_{1}\left(t\right)-L\frac{\int_{0}^{t}h\left(\tau \right)\sigma \left(\tau \right)d\tau }{V_{B0}\sigma \left(t\right)}\\ & =\left(\bar{A}-\frac{L}{{\bar{V}}_{B0}}\left[\begin{array}{ccc} 1 & 0 & 0 \end{array}\right]\right)e\left(t\right)+\eta \left(t\right) \end{array}$$

where $\eta \left(t\right)=\tilde{A}x\left(t\right)+\tilde{B}u\left(t\right)-\frac{L}{{\tilde{V}}_{B0}}x_{1}\left(t\right)-L\frac{\int_{0}^{t}h\left(\tau \right)\sigma \left(\tau \right)d\tau }{V_{B0}\sigma \left(t\right)}\left(t\right)$ is unknown disturbance (note that, here, we assumed that $\frac{1}{V_{B0}}=\frac{1}{{\bar{V}}_{B0}}+\frac{1}{{\tilde{V}}_{B0}}$).

To garner meaningful insights on how $e_{1}\left(t\right)$, $e_{2}\left(t\right)$, and $e_{3}\left(t\right)$ behave as well as to derive a metric for the detection of internal hemorrhage, we assumed that parametric uncertainty is small (i.e., $\tilde{A}x\left(t\right)+\tilde{B}u\left(t\right)-\frac{L}{{\tilde{V}}_{B0}}x_{1}\left(t\right)=0$), which leads to $\eta \left(t\right)\approx -L\frac{\int_{0}^{t}h\left(\tau \right)\sigma \left(\tau \right)d\tau }{V_{B0}\sigma \left(t\right)}\left(t\right)≜-L\eta_{h}\left(t\right)$. Expanding Equation (6) under this assumption yields the following expressions for the dynamics of individual errors:

(7) $$\begin{array}{c} {\dot{e}}_{1}\left(t\right)=-\left(k+\frac{l_{1}}{{\bar{V}}_{B0}}\right)e_{1}\left(t\right)+e_{2}\left(t\right)-e_{3}\left(t\right)-l_{1}\eta_{h}\left(t\right)\\ {\dot{e}}_{2}\left(t\right)=-\frac{l_{2}}{{\bar{V}}_{B0}}e_{1}\left(t\right)-\frac{k}{1+\alpha_{h}}e_{3}\left(t\right)-l_{2}\eta_{h}\left(t\right)\\ {\dot{e}}_{3}\left(t\right)=-\frac{l_{3}}{{\bar{V}}_{B0}}e_{1}\left(t\right)-l_{3}\eta_{h}\left(t\right) \end{array}$$

Deriving the transfer functions from $\eta_{h}\left(t\right)$ to $e_{1}\left(t\right)$, $e_{2}\left(t\right)$, and $e_{3}\left(t\right)$ yields the following:

(8) $$\begin{array}{c} e_{1}\left(s\right)=-\frac{l_{1}s^{2}+\left(l_{2}-l_{3}\right)s-\frac{k}{1+\alpha_{h}}l_{3}}{s^{3}+\left(k+\frac{l_{1}}{{\bar{V}}_{B0}}\right)s^{2}+\left(\frac{l_{2}}{{\bar{V}}_{B0}}-\frac{l_{3}}{{\bar{V}}_{B0}}\right)s-\frac{k}{1+\alpha_{h}}\frac{l_{3}}{{\bar{V}}_{B0}}}\eta_{h}\left(s\right)\\ e_{2}\left(s\right)=-\frac{l_{2}s^{2}+\left(kl_{2}-\frac{k}{1+\alpha_{h}}l_{3}\right)s-\frac{k^{2}}{1+\alpha_{h}}l_{3}}{s^{3}+\left(k+\frac{l_{1}}{{\bar{V}}_{B0}}\right)s^{2}+\left(\frac{l_{2}}{{\bar{V}}_{B0}}-\frac{l_{3}}{{\bar{V}}_{B0}}\right)s-\frac{k}{1+\alpha_{h}}\frac{l_{3}}{{\bar{V}}_{B0}}}\eta_{h}\left(s\right)\\ e_{3}\left(s\right)=-\frac{l_{3}s^{2}+kl_{3}s}{s^{3}+\left(k+\frac{l_{1}}{{\bar{V}}_{B0}}\right)s^{2}+\left(\frac{l_{2}}{{\bar{V}}_{B0}}-\frac{l_{3}}{{\bar{V}}_{B0}}\right)s-\frac{k}{1+\alpha_{h}}\frac{l_{3}}{{\bar{V}}_{B0}}}\eta_{h}\left(s\right) \end{array}$$

Note that, since $h\left(t\right)\geq 0$ and $\sigma \left(t\right)>0$, ∀t≥0, $\eta_{h}\left(t\right)\geq 0$, ∀t≥0. In addition, $\eta_{h}\left(t\right)$ monotonically increases through time during hemorrhage (i.e., when $h\left(t\right)>0$) as follows:

(9) $${\dot{\eta }}_{h}\left(t\right)=\frac{d}{dt}\left[\frac{\int_{0}^{t}h\left(\tau \right)\sigma \left(\tau \right)d\tau }{V_{B0}\sigma \left(t\right)}\right]=\frac{1}{V_{B0}}\left(h\left(t\right)-\frac{\dot{\sigma }\int_{0}^{t}h\left(\tau \right)\sigma \left(\tau \right)d\tau }{\sigma^{2}}\right)\geq \frac{1}{V_{B0}}h\left(t\right)$$

since $\dot{\sigma }\left(t\right)\leq 0$ during hemorrhage (i.e., HCT monotonically decreases as red blood cells lost due to hemorrhage are not replenished by the interstitial fluid exchange and/or resuscitation with fluid). Hence, $\eta_{h}\left(t\right)\geq \frac{1}{V_{B0}}\int_{0}^{t}h\left(\tau \right)d\tau$. To derive a condition for detecting internal hemorrhage, we also assumed that $h\left(t\right)$ varies slowly: $h\left(t\right)\approx H$, where H denotes a constant hemorrhage rate. Then, we inputted $\eta_{h}\left(s\right)\geq \frac{H}{V_{B0}s^{2}}$ into Equation (8) to derive the following terminal behaviors pertaining to $e_{1}\left(t\right)$, $e_{2}\left(t\right)$, and $e_{3}\left(t\right)$ using the final value theorem:

(10) $$\begin{array}{c} \underset{t\to \infty }{\lim}e_{1}\left(t\right)=\underset{s\to 0}{\lim}se_{1}\left(s\right)=\infty \\ \underset{t\to \infty }{\lim}e_{2}\left(t\right)=\underset{s\to 0}{\lim}se_{2}\left(s\right)=\infty \\ \underset{t\to \infty }{\lim}e_{3}\left(t\right)=\underset{s\to 0}{\lim}se_{3}\left(s\right)\geq \left(1+\alpha_{h}\right)H \end{array}$$

The last inequality in Equation (10) leads to the following condition:

(11) $$e_{3}\left(\infty \right)=x_{3}\left(\infty \right)-{\hat{x}}_{3}\left(\infty \right)\geq \left(1+\alpha_{h}\right)H\;\to \;{\hat{x}}_{3}\left(\infty \right)\leq x_{3}\left(\infty \right)-\left(1+\alpha_{h}\right)H$$

In words, Equation (11) implies that, in the steady state, ${\hat{x}}_{3}\left(t\right)$ is smaller than $x_{3}\left(t\right)$ when hemorrhage is present. In the absence of internal hemorrhage, $x_{3}\left(t\right)=h\left(t\right)=0$. Hence, when the parametric uncertainty is negligible, internal hemorrhage may be detected if ${\hat{x}}_{3}\left(t\right)\leq 0$. However, since the parametric uncertainty induces errors in ${\hat{x}}_{3}\left(t\right)$, a conservative condition must be used to suppress false positives. Hence, we arrived at the following condition for detecting internal hemorrhage:

(12) $${\hat{x}}_{3}\left(t\right)\leq {\underline{x}}_{3,LO}\left(t\right)$$

where ${\underline{x}}_{3,LO}\left(t\right)$ is the lower bound of $x_{3}\left(t\right)$ when $h\left(t\right)=0$, ∀t≥0 (see Section 2.3.1 for the details of how ${\underline{x}}_{3,LO}\left(t\right)$ is derived).

Beyond the detection of internal hemorrhage, the LO can also infer an approximate upper bound of its rate. Assuming that the rate of internal hemorrhage is constant (at H), we reduced Equation (11) to $H\leq -\frac{{\hat{x}}_{3}\left(\infty \right)}{\alpha_{H}}$. This inequality implies that the upper bound of the internal hemorrhage rate can be estimated from ${\hat{x}}_{3}\left(\infty \right)$ as $-\frac{{\hat{x}}_{3}\left(\infty \right)}{\alpha_{H}}$.

#### 2.2.2. Extended Kalman Filtering (EKF)

To design an EKF, we augmented two additional states to Equation (1): (i) $h\left(t\right)$ as an additional slowly varying state as $x_{3}\left(t\right)$ and (ii) the red blood cell volume as $x_{4}\left(t\right)$. Then, the resulting state–space representation is given by Equation (13):

(13) $$\begin{array}{c} \dot{x}\left(t\right)=f\left(x\left(t\right),u\left(t\right)\right)=\left[\begin{array}{c} -kx_{1}\left(t\right)+kx_{2}\left(t\right)-x_{3}\left(t\right)+u\left(t\right)\\ -\frac{1}{1+\alpha_{h}}x_{3}\left(t\right)+\frac{1}{1+\alpha_{u}}u\left(t\right)\\ 0\\ -\frac{{\;x}_{3}\left(t\right)x_{4}\left(t\right)}{{\;x}_{1}\left(t\right)+V_{B0}} \end{array}\right]\\ y\left(t\right)=h\left(x\left(t\right)\right)=\sigma \left(t\right)=\frac{x_{4}\left(t\right)}{{\;x}_{1}\left(t\right)+V_{B0}} \end{array}$$

**Remark 1.**  *One can imagine that an EKF may be designed based on Equation (4), where the output* $y\left(t\right)$ *is a nonlinear function of* $x_{1}\left(t\right)$*,* $h\left(t\right)$*, and* $\sigma \left(t\right)$: $y\left(t\right)=\frac{1}{V_{B0}}x_{1}\left(t\right)+\frac{\int_{0}^{t}h\left(\tau \right)\sigma \left(\tau \right)d\tau }{V_{B0}\sigma \left(t\right)}$*. However, this output equation is non-Markov, which presents challenges in designing an EKF.*

In the design of an EKF, we considered the process noise $w\left(t\right)$ and the sensor noise $v\left(t\right)$:

(14) $$\begin{array}{c} \dot{x}\left(t\right)=f\left(x\left(t\right),u\left(t\right),\theta \right)+w\left(t\right)\\ y\left(t\right)=h\left(x\left(t\right)\right)+v\left(t\right) \end{array}$$

where $\theta =\left\{V_{B0},\alpha_{u},\alpha_{h},k\right\}$. Instead of optimizing the process noise covariance, we designed the process noise $w\left(t\right)$ as the uncertainty in the state due to the parametric uncertainty:

(15) $$w\left(t\right)=J_{\theta }\left(t\right)\delta \theta \left(t\right),\;\;Q_{w}\left(t\right)=J_{\theta }\left(t\right)Q_{\theta }J_{\theta }^{T}\left(t\right)$$

where $J_{\theta }\left(t\right)$ is the Jacobian matrix of $f\left(x\left(t\right),u\left(t\right),\theta \right)$ with respect to θ, while $Q_{w}\left(t\right)$ and $Q_{\theta }$ are the process noise covariance matrix and the covariance matrix pertaining to θ, respectively (see Section 2.3.1 for the details of how $Q_{\theta }$ is derived). On the other hand, we designed the sensor noise $v\left(t\right)$ and its covariance $Q_{v}$ based on the noise associated with the measurement of $\sigma \left(t\right)$. Then, we estimated the states using the standard EKF prediction and correction procedure. At the time instant $t_{k}$, when a new measurement $\sigma \left(t_{k}\right)$ becomes available, the prediction procedure is given by:

(16) $$\begin{array}{c} \hat{x}\left(t_{k}|t_{k-1}\right)=\hat{x}\left(t_{k-1}\right)+\int_{t_{k-1}}^{t_{k}}f\left(\hat{x}\left(t\right),u\left(t\right),\theta \right)dt\\ P\left(t_{k}|t_{k-1}\right)=P\left(t_{k-1}\right)+\int_{t_{k-1}}^{t_{k}}F\left(t\right)P^{T}\left(t\right)+P\left(t\right)F^{T}\left(t\right)+Q_{w}\left(t\right)dt \end{array}$$

where $F\left(t\right)={\left.\frac{\partial f\left(x\left(t\right),u\left(t\right),\theta \right)}{\partial x}\right|}_{\hat{x}\left(t|t_{k-1}\right),u\left(t\right)}$. The correction procedure is given by:

(17) $$\begin{array}{c} K\left(t_{k}\right)=P\left(t_{k}|t_{k-1}\right)H^{T}\left(t_{k}\right){\left[H\left(t_{k}\right)P\left(t_{k}|t_{k-1}\right)H^{T}\left(t_{k}\right)+Q_{v}\left(t_{k}\right)\right]}^{-1}\\ \hat{x}\left(t_{k}\right)=\hat{x}\left(t_{k}|t_{k-1}\right)+K\left(t_{k}\right)\left[y\left(t_{k}\right)-h\left(\hat{x}\left(t_{k}|t_{k-1}\right)\right)\right]\\ P(t_{k})=\left[I-K\left(t_{k}\right)H\left(t_{k}\right)\right]P\left(t_{k}|t_{k-1}\right)\left[I-H^{T}\left(t_{k}\right)K^{T}\left(t_{k}\right)\right]+K\left(t_{k}\right)Q_{v}\left(t_{k}\right)K^{T}\left(t_{k}\right) \end{array}$$

where $H(t_{k})={\left.\frac{\partial h\left(x\right)}{\partial x}\right|}_{\hat{x}\left(t_{k}|t_{k-1}\right)}$.

In the EKF, ${\hat{x}}_{3}\left(t\right)$ is the estimate of internal hemorrhage. In the absence of internal hemorrhage, $x_{3}\left(t\right)=h\left(t\right)=0$. Hence, when the parametric uncertainty is negligible, internal hemorrhage may be detected if ${\hat{x}}_{3}\left(t\right)\geq 0$. However, since the parametric uncertainty induces errors in ${\hat{x}}_{3}\left(t\right)$, a conservative condition must be used to suppress false positives. Hence, we arrived at the following condition for detecting internal hemorrhage:

(18) $${\hat{x}}_{3}\left(t\right)\geq {\bar{x}}_{3,EKF}\left(t\right)$$

where ${\bar{x}}_{3,EKF}\left(t\right)$ is the upper bound of $x_{3}\left(t\right)$ when $h\left(t\right)=0$, ∀t≥0 (see Section 2.3.1 for the details of how ${\bar{x}}_{3,EKF}\left(t\right)$ is derived).

### 2.3. In Silico Evaluation

We conducted an array of extensive in silico investigations to evaluate and compare the efficacy of the two alternative sequential inference algorithms for the detection of internal hemorrhage and the estimation of its rate.

#### 2.3.1. Virtual Patients and Scenarios

As a basis to perform in silico evaluation, we used a mathematical model to replicate physiological responses to hemorrhage and resuscitation and its corresponding virtual patient generator (VPG) developed in our prior work [24]. In brief, we derived the mathematical model using 28 sheep, which underwent acute hemorrhage and resuscitation of fluids [25,26,27]. Then, we derived the VPG to be used in this work using the subset of the sheep resuscitated with colloid (*n* = 5) based on a collective variational inference (C-VI) method developed by us [24]. The VPG can generate random samples containing the parameters of the mathematical model (called the VPs), which allow the mathematical model to simulate physiologically plausible behaviors.

In this work, we conducted the in silico evaluation by sampling random VPs from the VPG and simulating the LO and the EKF on the VPs (as described in detail in Section 2.3.2). In each simulation, each VP was subject to a constant rate of internal hemorrhage and another constant rate of resuscitation with fluid after a brief period to settle down transients due to initial conditions. We considered the internal hemorrhage rate ranging 0.01–0.1 lpm and the resuscitation rate ranging 10–190% of the given internal hemorrhage rate in each VP. To examine the impact of the HCT error on the performance of detecting internal hemorrhage and estimating its rate, we considered the HCT error levels of 0%, 1%, 2%, and 3% in the form of Gaussian noise. Noting that the existing literature indicates that the current technology may achieve the HCT error of up to 3% [28], our investigation intends to garner insights on the performance of our sequential inference algorithms now and in the future when the measurement technology is further advanced. In sum, these combinations of hemorrhage rate, resuscitation rate, and HCT noise provided us with a total of 400 in silico evaluation scenarios: 10 (internal hemorrhage rates, from 0.01 lpm to 0.1 lpm in an increment of 0.01 lpm) × 10 (resuscitation rates, from 10% of hemorrhage rate to 190% of hemorrhage rate in an increment of 20%) × 4 (HCT noise levels) = 400. To balance true and false events in the in silico evaluation, we augmented to these 400 scenarios the same 400 scenarios but without hemorrhage. All in all, each VP underwent these 800 in silico evaluation scenarios, resulting in a total of 80,000 in silico evaluations. In each simulation, we ran the LO in Section 2.2.1 and the EKF in Section 2.2.2 to detect internal hemorrhage and estimate its rate (if any) as explained in Section 2.3.2.

#### 2.3.2. Internal Hemorrhage Detection and Hemorrhage Rate Estimation Based on Continuous Hematocrit Measurement

We implemented the LO and the EKF using the most likely (i.e., population average) parameter values in the VPG. In the case of the EKF, we used the covariance matrix pertaining to the VPG (which is derived by the C-VI method to represent the uncertainty associated with the VPs [24]) as $Q_{\theta }$ in Equation (15). We designed $Q_{v}$ as the HCT noise variance used in the in silico evaluation.

We specified the threshold values ${\underline{x}}_{3,LO}\left(t\right)$ in Equation (12) and ${\bar{x}}_{3,EKF}\left(t\right)$ in Equation (18) before conducting the in silico evaluations. For this purpose, we sampled 100 random VPs and simulated the LO and the EKF on them under all the 800 scenarios described in Section 2.3.1. Considering that the threshold values represent the lower bound of $x_{3}\left(t\right)$ estimated by the LO and the upper bound of $x_{3}\left(t\right)$ estimated by the EKF in the absence of internal hemorrhage, we conducted the simulations by applying the resuscitation input in the scenarios alone (i.e., we set the hemorrhage rate to zero in all these simulations). Then, we specified ${\underline{x}}_{3,LO}\left(t\right)$ as the minimum value of ${\hat{x}}_{3}\left(t\right)$ inferred by the LO across 80,000 simulations and we likewise specified ${\bar{x}}_{3,EKF}\left(t\right)$ as the maximum value of ${\hat{x}}_{3}\left(t\right)$ inferred by the EKF across 80,000 simulations.

Using the values of ${\underline{x}}_{3,LO}\left(t\right)$ and ${\bar{x}}_{3,EKF}\left(t\right)$ thus specified, we conducted the in silico evaluation. In this context, we sampled an additional 100 random VPs and simulated the LO and the EKF on them under all the 800 scenarios described in Section 2.3.1. The simulation was performed until (i) the VP lost >25% BV or (ii) the HCT of the VP was <10%. In each in silico evaluation (pertaining to a scenario and a VP), the LO and the EKF estimated their respective $x_{3}\left(t\right)$. In the case of LO, ${\hat{x}}_{3}\left(t\right)$ was compared with ${\underline{x}}_{3,LO}\left(t\right)$. In the case of EKF, ${\hat{x}}_{3}\left(t\right)$ was compared with ${\bar{x}}_{3,EKF}\left(t\right)$. Then, at each sampling time instant, the LO declared the detection of internal hemorrhage if Equation (12) was satisfied in >50% of the comparisons within the past 10 min window. Similarly, at each sampling time instant, the EKF declared the detection of internal hemorrhage if Equation (18) was satisfied in >50% of the comparisons within the past 10 min window. The 10 min window was used to mitigate the misdetection of internal hemorrhage due to fluctuations in ${\hat{x}}_{3}\left(t\right)$ caused by parametric uncertainty and measurement noise. Note that the use of the 10 min window may compromise the speed of detection. Hence, it can be viewed as the reconciliation between performance and robustness associated with the detection of internal hemorrhage. The LO and the EKF also estimated the rate of internal hemorrhage. In the case of LO, the upper bound of the rate of internal hemorrhage was estimated as $H\leq -\frac{{\hat{x}}_{3}\left(t\right)}{\alpha_{H}}$ (see Section 2.2.1). In the case of EKF, the rate of internal hemorrhage was estimated simply as ${\hat{x}}_{3}\left(t\right)$.

For both the LO and the EKF, we classified a detection declaration as “positive” if the internal hemorrhage was detected before (i) >25% of initial BV was lost or (ii) HCT was <10%. Otherwise, we classified the detection declaration as “negative”. The rationale behind this classification was to penalize the detections that were too late to save the lives of hemorrhaging patients.

To evaluate the performance of the sequential inference algorithms in comparison with the direct detection of internal hemorrhage based on the change in HCT, we evaluated the performance of a naïve algorithm, which declared the detection of internal hemorrhage if HCT decreased by >10% from its initial value:

(19) $$\sigma \left(t\right)\leq 0.9\times \sigma \left(0\right)$$

#### 2.3.3. Evaluation Metrics

To measure the accuracy of hemorrhage detection, we used the F1 score and the custom-defined corrected F1 score as primary metrics. The F1 score and the corrected F1 score differ in terms of how positives (i.e., hemorrhage detected) and negatives (i.e., hemorrhage not detected) are defined. In calculating the F1 score, we used the definitions of positive vs. negative detection described in Section 2.3.2; we defined a positive detection if, at any time instant in the evaluation, (i) the detection condition (Equation (12) for the LO and Equation (18) for the EKF) was satisfied in >50% of the comparisons within the past 10 min window and (ii) BV loss was ≤25% and HCT was ≥10%. We defined a negative detection otherwise if the detection condition was never satisfied until the evaluation ended or, equivalently, if the detection was too delayed (i.e., after >25% BV loss or <10% HCT). In this way, a positive detection can be obtained if its two requisite criteria are met at any time instant regardless of whether or not they are met afterwards. On the other hand, in calculating the corrected F1 score, we defined a positive detection if, at a sampling time instant, (i) the detection condition (Equation (12) for the LO and Equation (18) for the EKF) was satisfied in >50% of the comparisons within the past 10 min window, (ii) BV loss was ≤25% and HCT was ≥10%, and (iii) the detection condition remained satisfied till the end of the evaluation. We defined a negative detection otherwise. In this way, the corrected F1 score used a more stringent notion of positive detection than its F1 counterpart. We calculated the F1 score and the corrected F1 score on a VP-by-VP basis (note that each BP was evaluated under 800 scenarios as described in Section 2.3.1). Then, we calculated the descriptive statistics of these metrics.

In addition to F1 score and corrected F1 score, we used (uncorrected and corrected) precision, recall, sensitivity, and specificity as secondary metrics. To measure the speed of hemorrhage detection, we devised the normalized detection time (NDT), which is defined as the time to detect hemorrhage divided by the time to lose 25% of BV (considering that 30% blood loss is known to be associated with the occurrence of decompensation [29]) or the time to reach 10% HCT, whichever occurs first.

We determined the statistical significance in the differences in all the aforementioned metrics using the paired *t*-test with Bonferroni correction for multiple comparisons.

To measure the accuracy of estimating the rate of internal hemorrhage, we used the normalized absolute error (NAE) between true vs. estimated hemorrhage rates. For a given evaluation, the NAE was calculated as the average value of the absolute differences between true vs. estimated hemorrhage rates during the last 10% of the evaluation (to exclude the artifacts due to the transient behaviors in the estimated hemorrhage rate) divided by true hemorrhage rate.

## 3. Results

**Figure 2:**
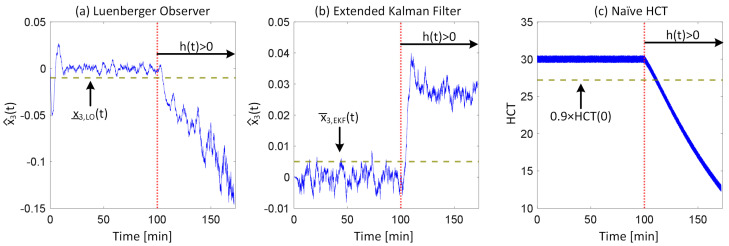
Representative examples of internal hemorrhage detection pertaining to (**a**) the LO (by Equation (12)), (**b**) the EKF (by Equation (18)), and (**c**) the naïve HCT (by Equation (19)) under 0.03 lpm hemorrhage rate, 0.015 lpm resuscitation rate, and 1% HCT noise.

**Figure 3:**
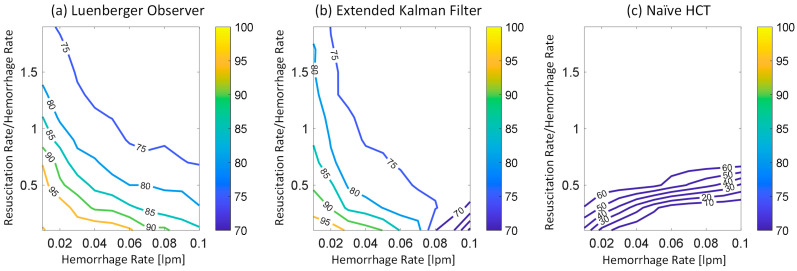
Average F1 score pertaining to (**a**) the LO, (**b**) the EKF, and (**c**) the naïve HCT across a wide range of internal hemorrhage and resuscitation rates. The average was taken over all the VPs.

**Figure 4:**
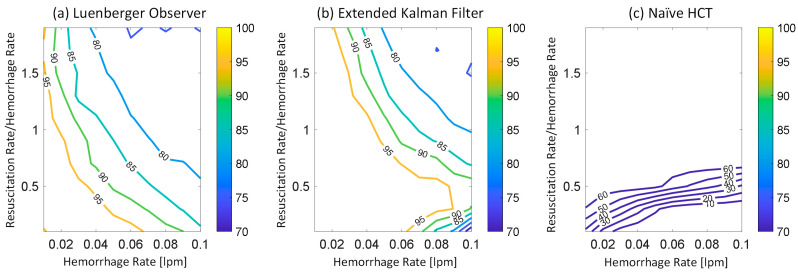
Average corrected F1 score pertaining to (**a**) the LO, (**b**) the EKF, and (**c**) the naïve HCT across a wide range of internal hemorrhage and resuscitation rates. The average was taken over all the VPs.

**Figure 5:**
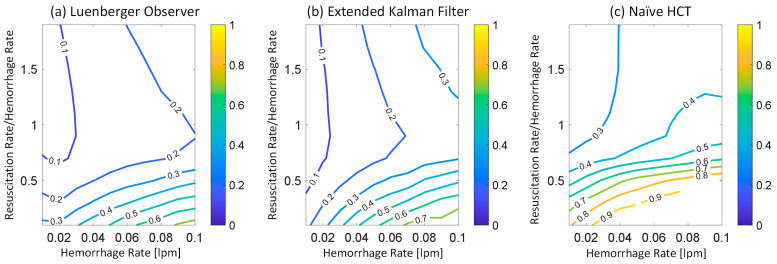
Average NDT pertaining to (**a**) the LO, (**b**) the EKF, and (**c**) the naïve HCT across a wide range of internal hemorrhage and resuscitation rates. The average was taken over all the VPs.

**Figure 6:**
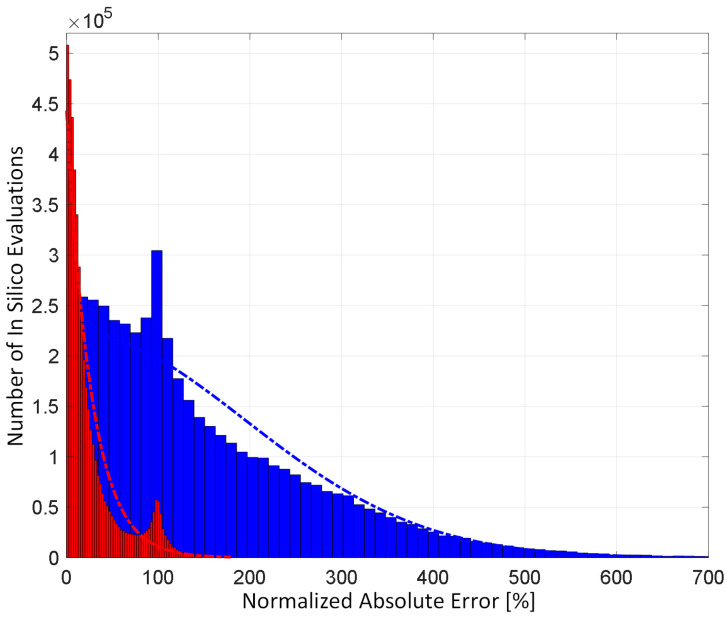
Probability distributions of errors associated with hemorrhage rate estimation pertaining to the LO and the EKF. The bars denote histograms. The dash dot lines denote the exponential distribution fitted to the histograms.

**Figure 7:**
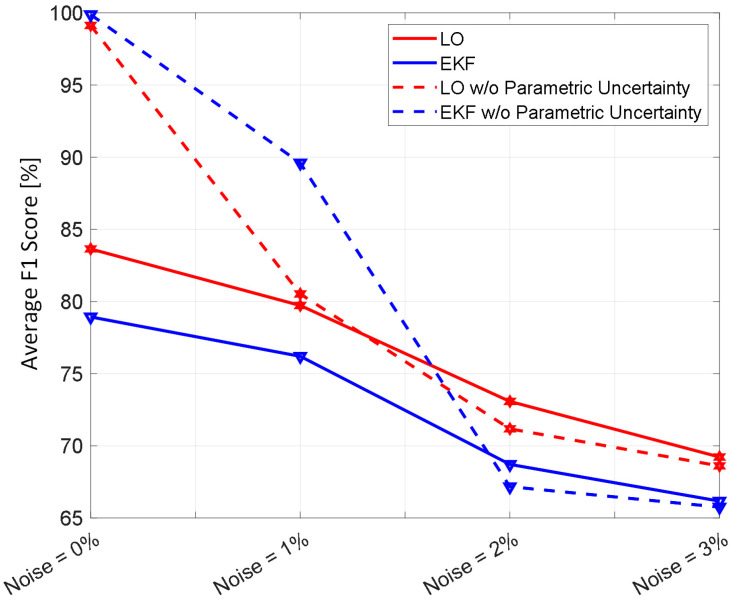
The impact of HCT measurement noise on the F1 scores pertaining to the LO and the EKF.

**Figure 8:**
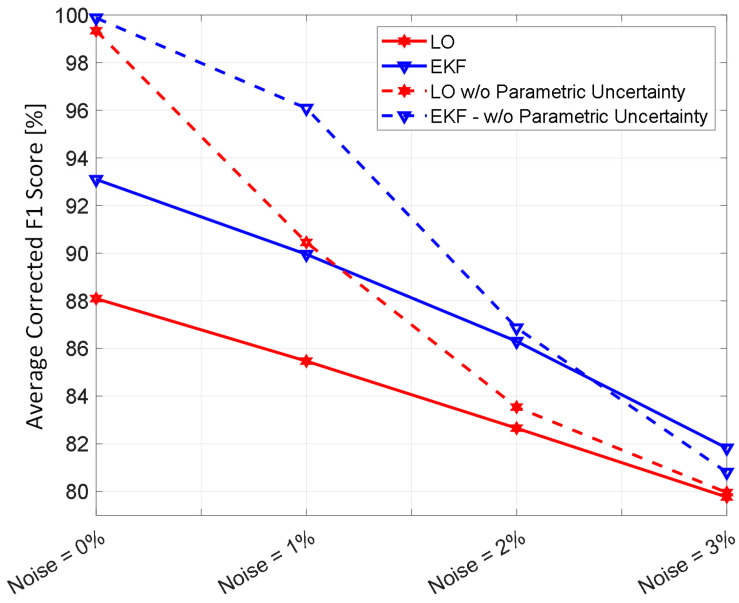
The impact of HCT measurement noise on the corrected F1 scores pertaining to the LO and the EKF.

**Figure 9:**
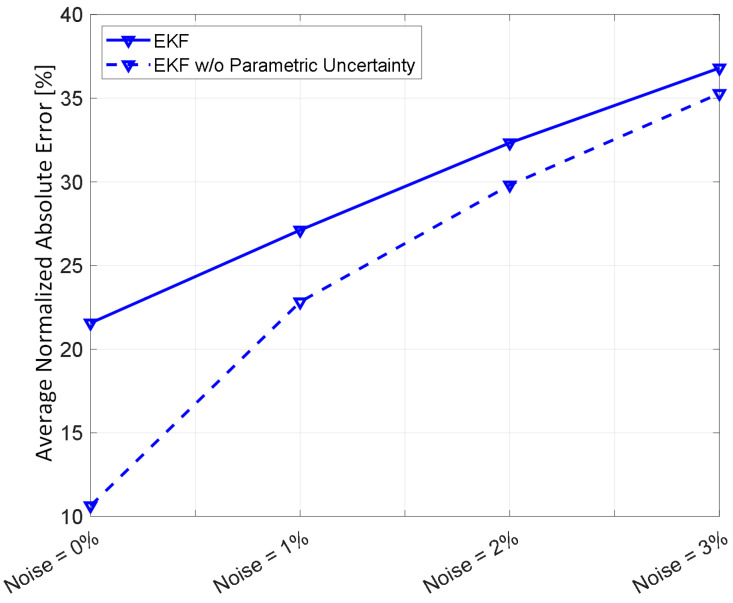
The impact of HCT measurement noise on hemorrhage estimation error pertaining to the EKF.

Figure 2 shows representative examples of internal hemorrhage detection pertaining to the LO (by Equation (12)), the EKF (by Equation (18)), and naïve HCT (by Equation (19)) under 0.03 lpm hemorrhage rate, 0.015 lpm resuscitation rate, and 1% HCT noise. Figure 3 and Figure 4 show the average F1 score and the average corrected F1 score, respectively, pertaining to the LO, the EKF, and the naïve HCT across a wide range of internal hemorrhage and resuscitation rates (note that the average was taken over all the VPs). Figure 5 shows the average NDT pertaining to the LO, the EKF, and naïve HCT across a wide range of internal hemorrhage and resuscitation rates (note that the average was likewise taken over all the VPs). Table 1 summarizes the statistics (in terms of mean and standard deviation) of precision, corrected precision, recall, sensitivity, specificity, accuracy, F1 score, corrected F1 score, and NDT pertaining to internal hemorrhage detection. Figure 6 shows the probability distributions of errors associated with hemorrhage estimation pertaining to the LO and the EKF. Figure 7 and Figure 8 show the impact of HCT measurement noise on the F1 scores and the corrected F1 scores pertaining to the LO and the EKF, respectively, while Figure 9 shows the impact of HCT measurement noise on hemorrhage estimation error pertaining to the EKF.

## 4. Discussion

Hemorrhage must be detected and treated promptly to save lives. However, prompt and accurate detection of internal hemorrhage is not feasible. Existing work to facilitate the detection of internal hemorrhage is often associated with a lack of interpretability, the detection of the decompensation threshold rather than hemorrhage itself, and/or the requirement for bulky equipment and trained operators. In this work, we investigated the proof-of-concept of detecting and estimating the rate of internal hemorrhage based on the sequential inference-enabled analysis of continuous noninvasive hematocrit measurement. In this context, we developed and evaluated two alternative sequential inference algorithms to detect internal hemorrhage and estimate its rate in an array of rigorous in silico evaluations. Primary goals of our work included (i) to investigate the potential of the sequential inference algorithms to enable continuous detection of internal hemorrhage, (ii) to garner in-depth insights on the behaviors (and, accordingly, strengths and weaknesses) of the sequential inference algorithms with respect to hemorrhage rate fluid resuscitation rates, (iii) to investigate the potential of the sequential inference algorithms to estimate the rate of internal hemorrhage, and (iv) to determine the impact of HCT measurement noise on the efficacy of the sequential inference algorithms. Details follow.

### 4.1. Sequential Inference Based on Continuous HCT for Detection of Internal Hemorrhage: Potential and Feasibility

The sequential inference has the potential to enable noninvasive hemorrhage detection superior to naïve HCT-based detection (Figure 2, Figure 3, Figure 4 and Figure 5, Table 1). The sequential inference (both the LO and the EKF) showed an F1 score of >0.7 and a corrected F1 score of >0.8 in many cases evaluated in this work, whereas both the F1 score and the corrected F1 score of naïve HCT-based detection were <0.5 (Figure 3 and Figure 4). In addition, the NDT pertaining to sequential inference was consistently smaller than NDT pertaining to naïve HCT-based detection (Figure 5).

In regards to hemorrhage detection, nonlinear (i.e., EKF) and linear (i.e., LO) sequential inference algorithms showed comparable performance (Figure 2, Figure 3, Figure 4 and Figure 5, Table 1). In many cases, both the LO and the EKF could detect hemorrhage promptly after hemorrhage occurred (Figure 2). As far as the F1 score and the corrected F1 score are concerned, the LO modestly outperformed the EKF in terms of the F1 score, while the EKF outperformed the LO in terms of the corrected F1 score. Scrutinizing the in silico evaluation results showed that the EKF was more prone to false detection (false positive) due to the transient fluctuations in the state estimates than the LO when internal hemorrhage and/or resuscitation started. However, most of the transient false detection events were associated with reasonably short durations (approximately <10% of the simulation time pertaining to the in silico evaluation scenario). Hence, such transient false alerts may not be too problematic. Further, transient false alerts due to resuscitation may be avoided by disabling the sequential inference algorithms for a brief period after resuscitation rate is adjusted. As far as the NDT is concerned, the LO and the EKF exhibited comparable performance. There was statistical significance in the difference in the NDT between them. But the absolute difference was small (3% on average; Table 1). All in all, internal hemorrhage detection performance was remarkable in both the LO and the EKF; the corrected F1 score was mostly >0.8 and, in the case of the EKF, >0.9 in approximately >50% of the scenarios investigated (Figure 4). The NDT was mostly <0.5, except in the region where hemorrhage rate is very high but resuscitation rate is very low (Figure 5).

### 4.2. Efficacy of Sequential Inference Algorithms with Respect to Hemorrhage and Fluid Resuscitation Rates

At the level of details, we note several interesting hemorrhage detection behaviors pertaining to sequential inference algorithms and naïve HCT-based detection. First, both the F1 score and the corrected F1 score pertaining to both the LO and the EKF tended to deteriorate as hemorrhage rate and resuscitation rate increased, although they were still good in the absolute sense (Figure 3 and Figure 4). This behavior may be attributed to the reasoning that the time available to detect internal hemorrhage decreases as hemorrhage rate and/or resuscitation rate increase(s) in most cases; BV is quickly lost if hemorrhage rate is high, while HCT quickly decreases if hemorrhage and/or resuscitation rates are high. However, also note that resuscitation restores the BV lost by internal hemorrhage and may improve the detection performance (and, accordingly, the F1 score and the corrected F1 score). Such a complex influence of resuscitation on the detection performance may explain the biphasic behavior of the corrected F1 score pertaining to the EKF, where it improved and then deteriorated as the resuscitation rate increased in the vicinity of 0.08 lpm hemorrhage rate (Figure 4). Second, both the F1 score and the corrected F1 score pertaining to naïve HCT-based detection were primarily governed by the resuscitation rate, although they were modestly governed by the internal hemorrhage rate as well; both the F1 score and the corrected F1 score tended to improve as resuscitation rate increased and hemorrhage rate decreased (Figure 3 and Figure 4). However, both the F1 score and the corrected F1 score tended to saturate when the resuscitation rate reached 40–60% of the hemorrhage rate. This behavior may be attributed to the reasoning that HCT quickly decreases as the resuscitation rate increases, which increases both true positives and false positives pertaining to naïve HCT-based detection (given that it declares detection due to the decrease in HCT regardless of the existence of internal hemorrhage). It appeared that both the F1 score and the corrected F1 score improved as true positives and false positives increased, after which they saturated when true positives and false positives (and, thus, true negatives) saturated. Third, the NDT pertaining to both the LO and the EKF as well as naïve HCT-based detection tended to improve as the resuscitation rate increased (Figure 5). This behavior may be attributed to the reasoning that resuscitation restores BV lost by hemorrhage, which extends the time to reach 25% BV loss.

### 4.3. Sequential Inference Based on Continuous HCT for Estimation of Internal Hemorrhage Rate: Potential and Feasibility

In contrast to hemorrhage detection, in regards to hemorrhage estimation, nonlinear sequential inference (i.e., EKF) significantly outperformed its linear counterpart (i.e., LO) (Figure 6). This observation is reasonable and anticipated because the EKF directly estimates hemorrhage rate, whereas the LO can only estimate the upper bound of hemorrhage rate (see Equation (11)). The hemorrhage estimation accuracy pertaining to the EKF may be practically useful: top 75% of NAE was <12%, top 50% of NAE was <7%, and top 25% of NAE was <3%.

### 4.4. Impact of HCT Measurement Noise on the Efficacy of Sequential Inference Algorithms

The analysis of the influence of sensor noise and plant parametric uncertainty on the detection and estimation of internal hemorrhage provided meaningful insights (Figure 7, Figure 8 and Figure 9). In regards to detection, parametric uncertainty dominated the detection performance when HCT measurement noise was small (<2%), while sensor noise dominated the detection performance when HCT measurement noise was large (≥2%) (Figure 7 and Figure 8). In regards to estimation of hemorrhage rate pertaining to the EKF, sensor noise but not parametric uncertainty dominated the estimation accuracy, and the trend appeared to be stronger as sensor noise level increased (Figure 9). Our analysis predicted that the corrected F1 score of ≥85% and ≥90% pertaining to the detection of internal hemorrhage may be achieved by the LO and the EKF, respectively, if sensor noise can be reduced to 1% level (from the currently available 3% level [28]). Our analysis also predicted that the accuracy of <30% pertaining to the estimation of the rate of internal hemorrhage may be achieved by the EKF if sensor noise can likewise be reduced to a 1% level. In addition, our analysis predicted that an additional 5–10% improvement may be achieved in the efficacy of both detecting and estimating the rate of internal hemorrhage if the sequential inference algorithms can be equipped with the ability to adapt to individual patients, e.g., by co-inferring the patient-specific mathematical model parameters together with the states. In this way, our analysis indicated that improving sensor accuracy and sequential inference algorithms may be equally important to improve hemorrhage detection and estimation performance.

## 5. Conclusions

We developed two alternative sequential inference algorithms based on continuous HCT sensing to enable prompt detection of internal hemorrhage. We showed that both the LO and the EKF can promptly detect internal hemorrhage across a wide range of hemorrhage and resuscitation rates. We also showed that the EKF can adequately estimate the rate of internal hemorrhage. The comparison of the sequential inference algorithms against a naïve HCT-based detection showed the superior performance of the sequential inference algorithms. All in all, our results provide supporting evidences to foster the future development of novel physics-based sequential inference analytics and high-accuracy high-precision continuous HCT sensing to mature internal hemorrhage monitoring capabilities.

## Figures and Tables

**Table 1:** Descriptive statistics (in mean and standard deviation) of precision, corrected precision, recall, sensitivity, specificity, accuracy, F1 score, corrected F1 score, and NDT pertaining to internal hemorrhage detection associated with the LO, the EKF, and naïve HCT-based detection, averaged over all the in silico evaluation scenarios (i.e., hemorrhage and resuscitation rates) under 1% HCT noise level. *: *p* < 0.016 with respect to naïve HCT-based detection. ^†^: *p* < 0.016 with respect to LO.

	LO	EKF	HCT
Precision	68 ± 13 *	63 ± 09 *^†^	45 ± 14
Corrected Precision	77 ± 14 *	85 ± 15 *^†^	45 ± 14
Recall	99 ± 03 *	98 ± 07 *	89 ± 29
Sensitivity	99 ± 03 *	99 ± 07 *	89 ± 29
Specificity	48 ± 24 *	39 ± 19 *^†^	0 ± 0
F1 Score	80 ± 08 *	76 ± 07 *^†^	60 ± 19
Corrected F1 Score	85 ± 08 *	90 ± 09 *^†^	59 ± 19
NDT	0.23 ± 0.14 *	0.26 ± 0.17 *^†^	0.33 ± 0.24

## Data Availability

The original contributions presented in the study are included in the article.
